# Effect of periostin on bone metabolic and autophagy factors during tooth eruption in mice

**DOI:** 10.1515/biol-2022-0663

**Published:** 2023-08-08

**Authors:** Han Qin, Jun Cai

**Affiliations:** Department of Stomatology, The Lianyungang Affiliated Hospital of Xuzhou Medical University, #182 Tongguan Road, Lianyungang 222002, Jiangsu Province, China; Department of Anesthesia, The Maternal and Child Health Hospital of Lianyungang City, Lianyungang 222006, Jiangsu Province, China

**Keywords:** periostin, tooth eruption, bone metabolic factors, autophagy factors

## Abstract

This study aimed to investigate the effect of periostin (PN) on the expression of receptor activator of nuclear factor-κB ligand (RANKL), osteoprotegerin (OPG), microtubule-associated protein1 light chain 3B (LC3B), and Beclin1 in mouse alveolar bone specimens and cultured osteoblasts *in vitro*, to preliminarily explore the role of PN and autophagy in remodeling bone metabolism during tooth eruption. Mice at 5 days of age were injected with 75 ng/mL recombinant PN protein under the periosteum for 3 consecutive days according to the standard of 1 mL/100 g/day. Then, their mandibles were removed, and the expression of bone metabolic and autophagy factors was detected by immunohistochemistry. Mouse osteoblast-like cells cultured *in vitro* were treated with recombinant PN at a concentration of 75 ng/mL. The changes in the aforementioned indicators were compared again by immunofluorescence and western blotting 72 h after dosing. The results of the mouse samples showed that the protein expression of RANKL, LC3B, and Beclin1 decreased, accompanied by the decrease in RANKL/OPG ratio. However, OPG protein expression increased in the dosing group. Immunofluorescence and western blotting results of osteoblasts cultured *in vitro* showed that the protein expression of RANKL, LC3B, Beclin1, and the RANKL/OPG ratio in the experimental group decreased, but OPG expression increased. PN may regulate alveolar bone metabolism during tooth eruption by inhibiting the RANKL/OPG ratio and autophagy, which will provide a new research perspective for further exploration of the mechanisms during tooth eruption.

## Graphical Abstract

**Figure:**
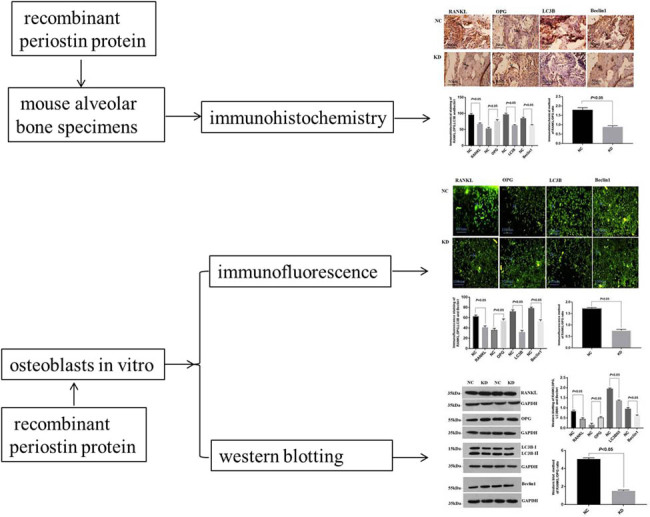

## 1 Introduction

Normal tooth eruption channel formation is a multifactorial and complex process, which requires coordinated differentiation of osteoclasts and osteoblasts to maintain the dynamic balance of alveolar bone [1]. The receptor activator of nuclear factor-κB ligand (RANKL)/receptor activator of NF-κβ (RANK)/osteoprotegerin (OPG) system is an important regulatory pathway of bone metabolism. Combinatorial RANKL in osteoblasts and RANK expressed on osteoclast precursor surfaces constitute the most critical signaling pathway for osteoclast activation. OPG acts as a decoy receptor to competitively block RANKL–RANK binding. Its main functions are to inhibit osteoclast differentiation and bone resorption activity of mature osteoclasts and promote the formation of bone matrix. In this way, OPG can maintain bone metabolic balance by regulating the signaling pathway of osteoblast/osteoclast differentiation. Therefore, the RANKL/OPG ratio can be used as a landmark index to measure normal bone metabolism [2,3,4].

As an important mechanism of cell self-protection, autophagy plays a decisive role in maintaining cell function and internal environmental stability. Recent studies have shown that autophagy genes are expressed in various stages of tooth development, which means that autophagy may be also involved in the formation of tooth eruption channels [5,6]. Our previous experimental results showed that the expression of RANKL protein in osteoblasts decreased with a decrease in autophagy function, suggesting that RANKL and autophagy may synergistically regulate osteoblast differentiation [7].

When periostin (PN) was first isolated from a mouse MC3T3-E1 osteoblast cell line, it was named as osteoblast-specific factor 2. With the continuous development of research, it has been found that PN is a multifunctional extracellular matrix protein mainly localized in the periodontal ligament and maintains the dynamic balance of alveolar bone metabolism. PN plays an important coordinating role in regulating periodontal tissue function, tooth development, and the regeneration of tissue repair caused by mechanical stress [8]. Our previous study used the same osteoclast-like cells as this experiment and found that the expression of autophagy proteins increased after PN gene silencing, suggesting that PN and autophagy may regulate the differentiation process of osteoblasts in a negative correlation [9]. This study focuses on the relationship between PN, RANKL/OPG ratio, and autophagy, how these factors coordinate alveolar bone remodeling during tooth eruption, and whether it can provide a valuable experimental basis for tooth eruption by discussing their mechanism.

## 2 Materials and methods

### 2.1 Immunohistochemical staining of mouse alveolar bone specimens

Ten mice born in the same litter at 5 days old, weighing 3.0–3.5 g, were randomly divided into group KD and group NC. All the mice were fed from their mothers, who were freely ingested water and feed, and were fed cleanly. Recombinant PN protein at the concentration of 75 ng/mL was injected into the alveolar bone of mice in group KD for 3 consecutive days according to the standard of 1 mL/100 g/day, while normal saline was injected into the mice alveolar bone in group NC. 3 days later, all mice were decapitated and euthanized. Then, each mouse mandible was fixed with 4% paraformaldehyde for 1 day and decalcified at 4°C in sodium formate decalcification solution for 3 days. After demineralization, the mandible was cut, pruned, dehydrated with ethanol, transparent with xylene, and embedded in paraffin to prepare 5 μM thick paraffin sections. All tissue sections were then dewaxed with water. The specimens were immunohistochemically stained on the basis of the kit manufacturer’s instructions. The primary antibodies used were mouse anti-RANKL (Proteintech Company, NO 66610-1-Ig, 1:50), rabbit anti-OPG (Abcam Company, NO ab9986, 1:50), rabbit anti-light chain 3B (LC3B) (Abcam Company, NO #ab51520, 1:100), and mouse anti-Beclin1 (CST Company, NO #4122, 1:100). The secondary antibody used was diluted anti-rabbit IgG (Santa Cruz Company, NO sc-2004, 1:400). After completion of the immunohistochemical reaction, hematoxylin was used for slight re-staining. Then, the gradient ethanol was dehydrated and made transparent with xylene. Protein expression was observed with a microscope after sections sealed with neutral glue.


**Ethical approval:** The research related to animal use has been complied with all the relevant national regulations and institutional policies for the care and use of animals.

### 2.2 Osteoblast culture and experimental grouping

Cryopreservation tubes of mouse osteoblast-like (MC3T3-E1) cells purchased from Shanghai Sangon Biotechnology Co., LTD, were taken out from the liquid nitrogen tank. After complete thawing and centrifugation at 1,300 rpm for 3 min, the supernatant was collected. Cells were resuscitated at 37°C in 5% CO_2_ incubator using the Eagle method with the minimum necessary medium and then cultured. The cell medium was changed every alternate day until the cell aggregation reached about 80%. The cells in the logarithmic growth stage were digested with pancreatic enzymes, and a 3–5 × 10^4^/mL single-cell suspension was prepared. Thereafter, the cell suspension was placed on pre-prepared sterilized coverslips in a 24-well culture plate. Previous literature and our preliminary experimental results have shown that the PN protein concentration is significant at 75 ng/mL. According to the experimental requirements, osteoblasts were assigned to the KD and NC groups. The group KD was added with 75 ng/mL recombinant PN protein prepared in the culture medium, while the group NC was changed to serum-free culture medium and cultured for 72 h.

### 2.3 Osteoblast immunofluorescence staining

After 72 h of dosing and culture, osteoblast climbing tablets were removed and set with 4% paraformaldehyde for 15 min. The samples were subjected to immunofluorescence staining, which was in line with the kit manufacturer’s instructions. The primary antibodies used were mouse anti-RANKL (Proteintech Company, NO 66610-1-Ig, 1:50), rabbit anti-OPG (Abcam Company, NO ab9986, 1:50), rabbit anti-LC3B (Abcam Company, NO #ab51520, 1:100), and mouse anti-Beclin1 (CST Company, NO #4122, 1:100). Diluted anti-rabbit IgG (Santa Cruz Company, NO sc-2004, 1:400) was used as the secondary antibody. After completion of the immunofluorescence reaction, 4′, 6-diamidino-2′-phenylindole (DAPI) was added drop-wise and incubated in the dark for 5 min, and the samples were stained for nuclei. Excess DAPI was washed four times with phosphate-buffered saline (PBS) for about 5 min each. The film was sealed with a solution containing an anti-fluorescence quenching agent, and the collected images were observed under a fluorescence microscope.

### 2.4 Osteoblast detection by western blotting

Osteoblasts were made into samples with or without the addition of exogenous PN proteins. When adherent cells reached the desired degree of growth integration, the culture medium was removed. The cells were washed twice with precooled PBS and then lysed in extraction buffer. According to the instructions of bicinchoninic acid protein assay kits, gels with different concentrations were prepared in accordance with the target protein molecular weight. Sodium dodecyl sulfate–polyacrylamide gel electrophoresis was then completed. After electrophoresis, the detected proteins were transferred to polyvinylidene fluoride (PVDF) membranes, at a constant current of 300 mA for 150 min at 4°C, by a transfer electrophoresis device. The diluted polyclonal antibody (primary antibody) mouse anti-RANKL (Proteintech Company, NO66610-1-Ig, 1:85, 35–38 kDa), rabbit anti-OPG (Abcam Company, NO ab9986, 1:100, 56 kDa), rabbit anti-LC3B (Abcam Company, NO#ab51520, 1:300, 14, 16 kDa), and mouse anti-Beclin1 (CST Company, NO#4122, 1:300, 60 kDa) were incubated with the blocked PVDF membrane overnight. After thorough cleaning, the PVDF membrane was treated with horseradish peroxidase-conjugated anti-mouse IgG antibody (CST Company, NO#7076, 1:2,000) or anti-rabbit IgG antibody (CST Company, NO#7074, 1:2,000) for 1.5 h at room temperature. Once again, the PVDF film was thoroughly cleaned, and chemiluminescent detection was performed using an enhanced chemiluminescence protein blotting substrate kit. Glyceraldehyde-3 phosphate dehydrogenase (Santa Cruz, NO#sc-32233, 1:2,000, 36 kDa) was selected as the loading control.

### 2.5 Statistical analysis

The experimental results were shown as the mean ± standard error of the mean. The *t*-test method in SPSS 19.0 statistical software was used for statistical analysis of the data, and *p* < 0.05 was considered as statistically significant.

## 3 Results

### 3.1 PN efficiency on bone metabolic and autophagy factors in mouse alveolar bone specimens

**Figure 1:**
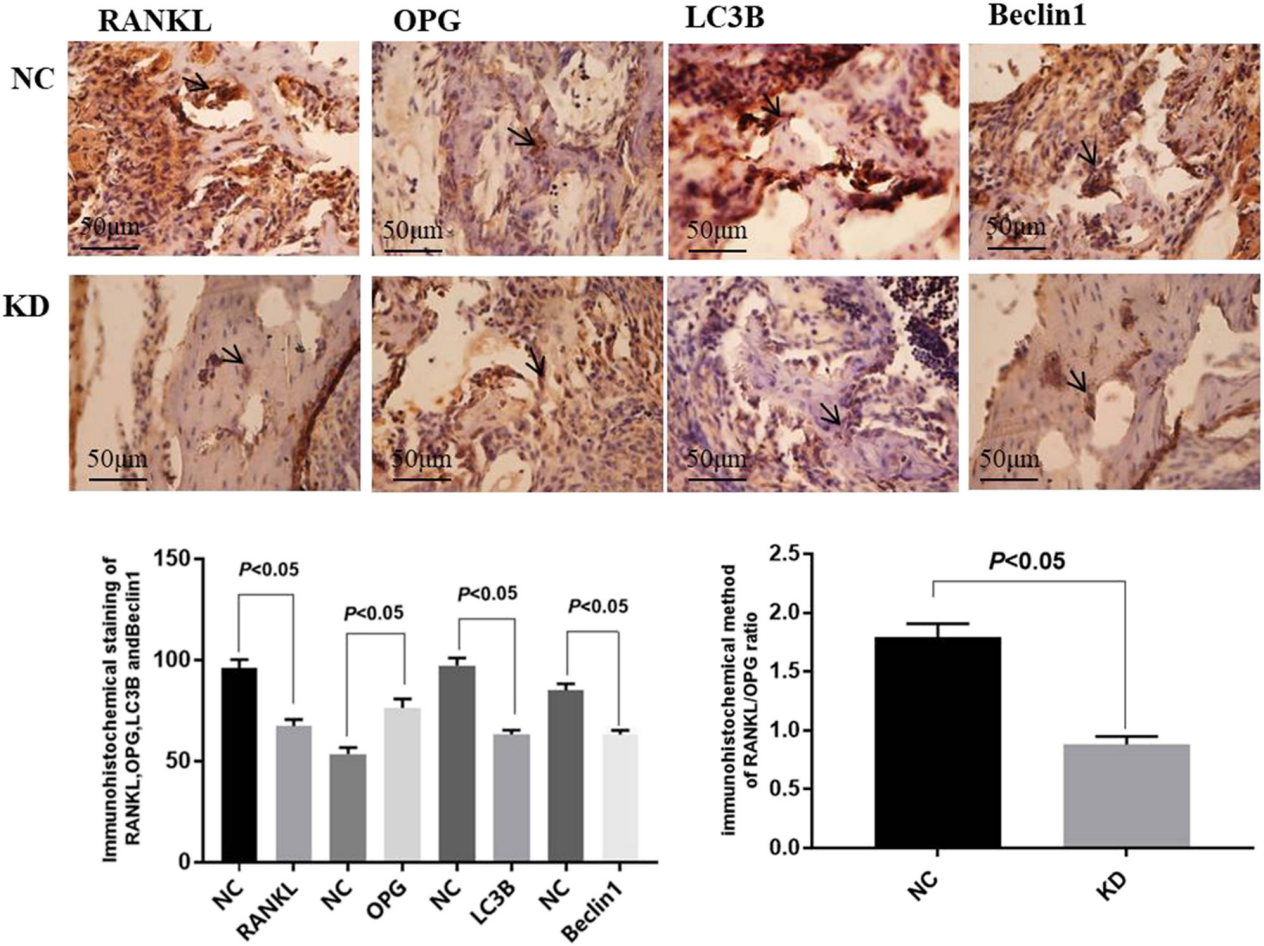
Immunohistochemical detection of alveolar bone suggested that the expression of RANKL, LC3B, and Beclin1 in group KD was weaker, while the expression of OPG in group KD was stronger than that in group NC (×200). The results of statistical analysis showed that the protein expression of RANKL, LC3B, and Beclin1, and the ratio of RANKL/OPG in group KD were lower, but OPG expression was higher than that in group NC (*p* < 0.05).

Ten mice born in the same litter at 5 days of age were randomly divided into the group KD and NC. Recombinant PN protein at a concentration of 75 ng/mL was injected into alveolar bone of the group KD for 3 days. Immunohistochemical staining of the mouse alveolar bone specimens was performed on the basis of the kit manufacturer’s instructions. Strong positive cells of RANKL, LC3B, and Beclin1 immunohistochemical staining were detected in the alveolar bone tissue of the group NC, showing dense and evenly distributed flake-brown precipitation. Immunohistochemical staining for RANKL, LC3B, and Beclin1 in the alveolar bone tissue of the group KD was weakly positive. However, the results of OPG immunohistochemical staining showed that the expression in group KD was higher than that in group NC. Statistical analysis of immunohistochemical results indicated that the optical density values of RANKL, LC3B, Beclin1, and the RANKL/OPG ratio in group KD were lower than those in group NC, while the optical density values of OPG were higher than those in group NC, all of which were statistically significant (Figure 1).

### 3.2 PN efficiency on autophagy and bone metabolic factors of osteoblasts cultured *in vitro*

**Figure 2:**
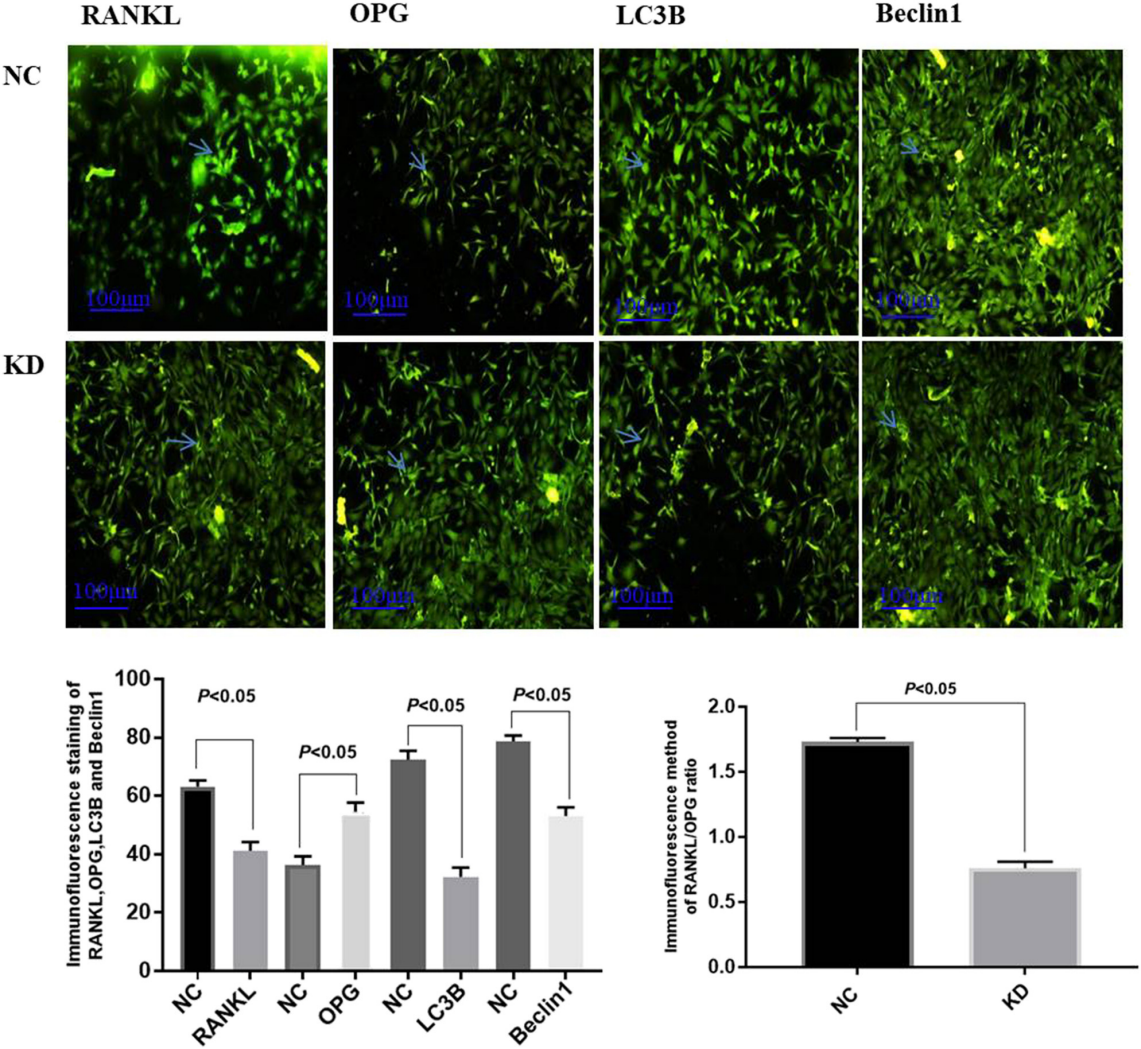
Immunofluorescence detection of osteoblasts suggested that the expression of RANKL, LC3B, and Beclin1 was weaker, while the expression of OPG was stronger in group KD (×100). The results of statistical analysis showed that the protein expression of RANKL, LC3B, and Beclin1, and the ratio of RANKL/OPG in group KD were lower, but OPG expression was higher than that in group NC (*p* < 0.05).

Regardless of whether the concentration of 75 ng/mL recombinant PN protein was added, all the osteoblasts presented with obvious pseudopodia, indicating that the cells grew well and had a strong activity at this time, which could be used for subsequent experimental studies. Immunofluorescence detection revealed weak RANKL, LC3B, and Beclin1 green fluorescence in osteoblasts treated with recombinant PN, and strong fluorescence was observed in group NC. But the expression intensity of OPG in group KD was higher than that in group NC. Five climbing films of groups KD and NC were randomly selected, and the RANKL-, OPG-, LC3B-, and Beclin1-positive cells of each climbing film were randomly chosen using Image Pro Plu software to measure the mean optical density value of six visual fields. The results suggested that compared with group NC, the optical density values of RANKL, LC3B, Beclin1,and the RANKL/OPG ratio decreased, while the optical density values of OPG increased in the group KD, all of which were statistically significant (Figure 2).

**Figure 3:**
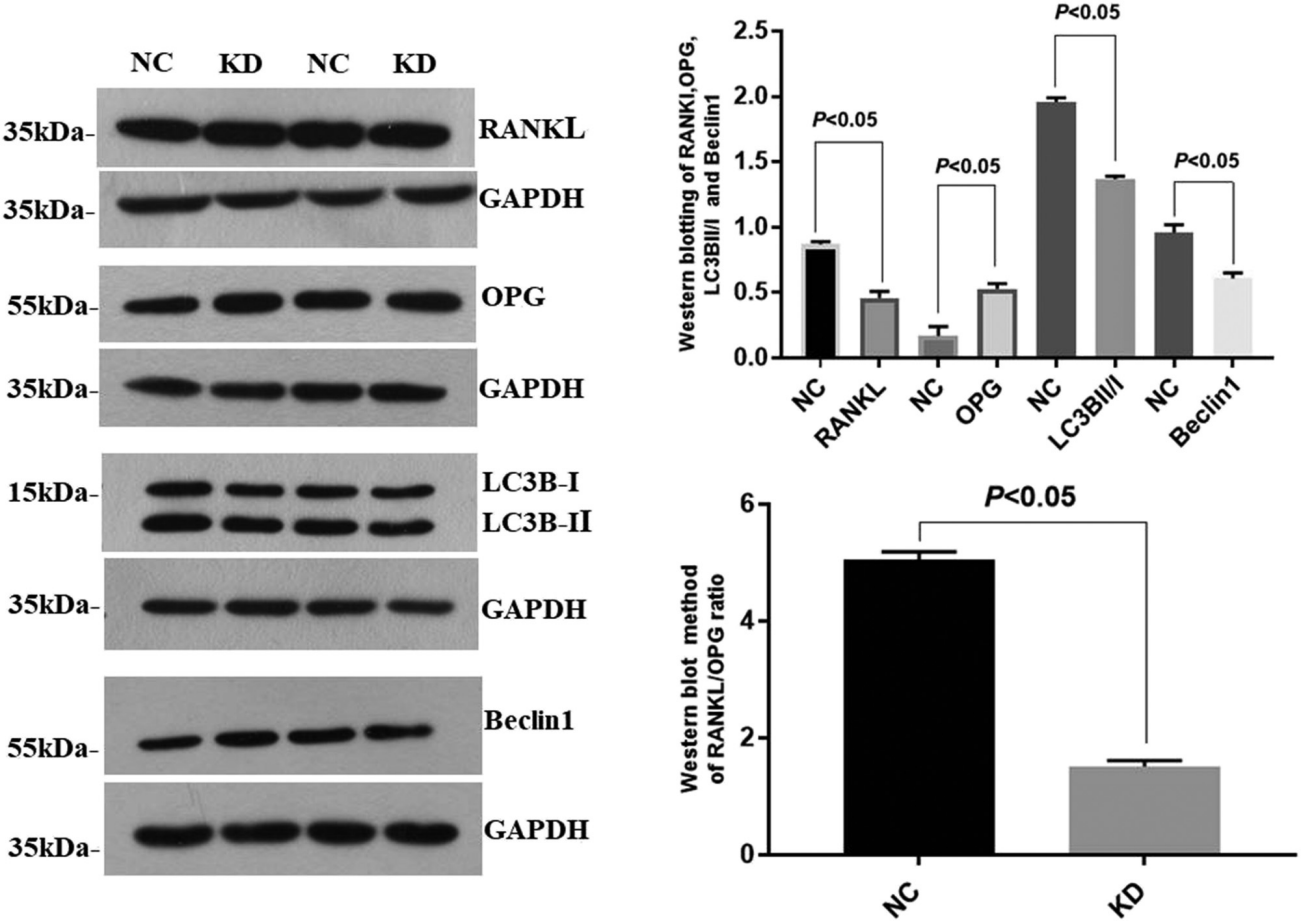
Western blotting detection of osteoblasts suggested that the expression of RANKL, LC3B, and Beclin1 in group KD was weaker, while the expression of OPG was stronger. The results of statistical analysis showed that the protein expression of RANKL, LC3B, and Beclin1, and the ratio of RANKL/OPG in group KD were lower, but OPG expression was higher than that in group NC (*p* < 0.05).

Western blotting results of osteoblasts treated with exogenous recombinant PN revealed that the protein expression of RANKL, LC3B, and Beclin1 was down-regulated in group KD; however, the OPG expression was up-regulated compared to that in group NC. Gel Pro analyzer software was used for gray value analysis; compared with group NC, the gray values of RANKL, LC3B, Beclin1, and RANKL/OPG ratio in group KD decreased, while the OPG gray value increased, all of which were statistically significant (Figure 3).

## 4 Discussion

Precise interaction between osteoclasts and osteoblasts is an important guarantee for ensuring teeth eruption to the correct position [10,11]. Previous studies on the mechanism of tooth eruption have mainly focused on the regulation of osteoclast-related molecular morphology and function. Few studies have investigated how osteoblasts initiate osteoclast differentiation and maintain bone homeostasis during tooth eruption pathways. As a cytokine isolated from the murine MC3T3-E1 osteoblastic cell line, PN was initially named as osteoblast-specific factor 2. Due to its special distribution in periosteum and periodontal ligament, as well as its priority and high expression in periosteum during embryogenesis and bone formation, it was later renamed PN [12]. Therefore, the purpose of this study was to investigate the influence of PN on the changes in osteoblast function and to better understand the theoretical basis for studying alveolar dynamic balance during tooth eruption.

It was found that PN expression in pre-osteoclasts and osteoclasts increased six-fold at the end of cell differentiation, suggesting that PN plays a critical role in bone remodeling [13]. Other findings suggest that PN is closely linked to bone metabolism genes, which is manifested by a decrease in osteoblast factor expression and an increase in osteoclast factor expression when PN gene is silenced [14]. Researchers have cultured periodontal ligament stem cells (PDLScs) from periodontitis patients accompanied with diabetic mellitus *in vitro* and found that exogenous periosteum proteins can reverse the osteogenic differentiation of PDLScs. This study suggests that PN proteins may play a promoting role in restoring cellular function under the inflammatory microenvironment [15]. Other studies have shown that the eruption of incisors in mice was disordered when the PN gene was knocked out. Histological observations revealed that the shear band disappeared, indicating that PN is an extracellular matrix protein necessary for the formation of tooth eruption channels in mice [16]. This suggests that PN may be a negative factor during tooth development and eruption, which regulates tooth eruption channels; however, the exact regulatory mechanism is still unclear.

The normal teeth eruption is a complicated and orderly physiological process regulated by many factors, involving the coordination of the tooth germ, alveolar bone, and a variety of cells and molecules [17,18]. The dynamic balance of osteoblast/osteoclast differentiation is crucial for sprouting channel formations. As a key signaling pathway for regulating bone homeostasis, the RANKL/RANK/OPG system plays a critical role in bone tissue formation, bone remodeling, and tooth eruption channel completion by regulating osteoclast differentiation signal pathway and bone matrix formation [19,20,21]. The osteoclast differentiation factor RANKL expressed by osteoblast combines with the receptor RANK expressed by osteoclast to accelerate differentiation and maturation of osteoclast. OPG, a decoy receptor, inhibits RANK–RANKL binding by competing with RANKL. Therefore, the RANKL/OPG ratio in the local microenvironment plays a defining role in the state of alveolar bone absorption [22,23].

The first mandibular molars of the mice erupted on the 15th day after birth. Alveolar bone metabolism was active on the 5th to 7th days after birth and then gradually slowed down on the 12th day [24,25]. Therefore, in this study, we selected mice on the 5th day after birth and injected recombinant PN protein into the alveolar bone continuously for 3 days. At this time, the index of osteoclast differentiation and metabolism reached a maximum, which could better reflect the experimental effect of the drug. In accordance with the results obtained from literature review and our preliminary experiments, recombinant PN protein was prepared at a concentration of 75 ng/mL with normal saline. Then, the drug is locally injected into the alveolar periosteum of the mouse molar area, and the expression of bone metabolic factors was observed. The results showed that after 75 ng/mL recombinant PN protein was added to the alveolar bone tissue of the mouse, the expression of the bone destruction factor RANKL protein decreased, while the expression of the bone formation factor OPG protein increased; thus, the RANKL/OPG ratio decreased. Similarly, the same concentration of recombinant PN protein was added to osteoblasts cultured *in vitro*, and the aforementioned indicators were detected once again. Results of immunofluorescence and western blotting both showed that after adding PN protein, the RANKL protein expression decreased and the OPG protein expression increased, which results in a lower RANKL/OPG ratio. In this way, the experimental results for mouse alveolar bone were verified by osteoblasts cultured *in vitro.* PN may inhibit bone resorption and promote bone formation by adjusting the change in the RANKL/OPG ratio in the local microenvironment to prevent alveolar bone excessive absorption and maintain the normal formation of tooth eruption channels.

Autophagy genes can be expressed in different stages of tooth development [26,27]. However, there are few studies on the role of autophagy in tooth eruption. Our previous studies have found that decreased autophagy function in osteoblasts may be a critical factor for tooth eruption channel disorders. In addition, a study of osteoblasts cultured *in vitro* showed that a decreased RANKL expression was accompanied by the decreased expression of autophagy factors, indicating that there may be a positive correlation between RANKL and autophagy [7]. Our other previous study found that the autophagy protein expression increased when the PN gene of bone cells was silenced, suggesting that PN was negatively correlated with autophagy [9]. This makes us think about the relationship between PN, the RANKL/OPG ratio, and autophagy during tooth eruption. Based on the aforementioned ideas, we detected autophagy-related indices of mouse alveolar bone tissue injected with recombinant PN protein and osteoblasts cultured *in vitro* with recombinant PN protein. Based on the results of *in vivo* and *in vitro* experiments, some meaningful conclusions were drawn as follows: (1) the mice at 5–7 days of age were in the active period of alveolar bone metabolism, accompanied by the enhancement of autophagy, which provides a certain reference for further study of autophagy function during mouse tooth eruption. (2) The appropriate concentration and action time of recombinant PN protein were selected, which provided reference for the study of the best effect of the PN protein. (3) Another way that PN protein inhibits alveolar bone resorption may be by inhibiting the autophagy function of osteoblasts, which will expand ideas for the clinical application of PN.

During the entire process of tooth eruption, the tissue around the tooth germ expresses various stimulating factors that promote the proliferation and differentiation of osteoclasts [28,29]. Therefore, there must be another negative regulator that maintains the dynamic balance of tooth eruption without excessive alveolar bone absorption [30,31]. As a new extracellular matrix protein that can promote the differentiation and adhesion of osteoblasts, PN plays a crucial role during tooth growth, development, and eruption [32,33]. Combined with previous research results, this study confirmed that PN may negatively regulate alveolar bone absorption during tooth eruption by inhibiting the RANKL/OPG ratio and autophagy function through *in vivo* animal experiments and *in vitro* osteoblast cultures. This opens up a new idea for interpreting the related molecular signal mechanism of PN in the process of tooth eruption to better understand the regulatory mechanism of bone mass dynamic balance during tooth eruption. This study provides a new research perspective for the application of exogenous PN protein in the treatment of abnormal tooth eruption, tooth and alveolar bone fractures, periapical lesions, periodontal diseases, etc.
